# A New Model Describing Copper Dose–Toxicity to Tomato and Bok Choy Growth in a Wide Range of Soils

**DOI:** 10.3390/ijerph16020264

**Published:** 2019-01-18

**Authors:** Bao Jiang, Yibing Ma, Guangyun Zhu, Jun Li

**Affiliations:** 1 Institute of Agricultural Resources and Regional Planning, Chinese Academy of Agricultural Sciences, Beijing 100081, China; jiangbao@caas.cn (B.J.); lijun01@caas.cn (J.L.); 2 School of Resources and Environment, University of Jinan, Jinan 250022, China; zhuguangyun1993@163.com; 3 Laboratory of Quality and Safety Risk Assessment for Microbial Products (Beijing), Ministry of Agriculture, Beijing 100081, China

**Keywords:** copper, dose–response relationship, EDTA-extractability, phytotoxicity

## Abstract

Phytotoxicity thresholds for heavy metals are derived from dose–response curves, which show the relationships between exposure dose and toxicity response. However, the results of tests or observations are commonly based on total heavy metal concentration, not the exposure dose that causes phytotoxicity; additionally, the phytotoxicity response differs with plant species. In the present study, the ethylenediaminetetraacetic acid (EDTA)-extractable copper (Cu) concentration was determined in order to evaluate Cu extractability. As two important horticultural food crops in Asia, tomato (*Lycopersicon esculentum* ‘Meifen No. 1’) and bok choy (*Brassica rapa* var. *chinensis* ‘Susheng 28’) were used to investigate Cu phytotoxicity in a wide range of Chinese soils with and without leaching treatment, after which relationships between Cu phytotoxicity thresholds based on EDTA-extractions and soil properties were established. The phytotoxicity thresholds showed that biomass of bok choy was more sensitive to Cu than tomato. Multiple linear regression analyses showed that soil factors, including organic carbon (OC), citrate dithionate extractable manganese (CD-Mn), cation exchange capacity (CEC), and CaCO_3_ explained over 85% of the variance in Cu phytotoxicity thresholds. The EDTA-extractable Cu dose–response relationships were further improved by incorporating soil properties. The new phytotoxicity predictive models indicated soil properties (soil pH, OC, CEC, and oxalate-extractable Mn) and EDTA-extractable Cu concentration explained more than 90% of the variance in the phytotoxicity response of tomato and bok choy biomass. The new phytotoxicity predictive models could be used to develop a reasonable remediation strategy for contaminated soils.

## 1. Introduction

Continuing accumulation of copper (Cu) in surface soils, particularly in agricultural lands, will increase the risk of phytotoxicity, and eventually threaten environment security [1]. Heavy metal accumulation in soils is of concern in agricultural production due to adverse effects on growth (due to phytotoxicity) and environmental health [2]. As an indispensable food group, vegetables play important roles in agricultural production. In recent years, research on the phytotoxicity of heavy metals to vegetables has attracted more attention. Yang et al. [3] found that for three vegetable crops (Chinese cabbage (*Brassica chinensis* L.), bok choy (*Brassica chinensis* L.), and celery (*Apiumg graveolens* L. *var. dulce* DC)) shoot growth was significantly inhibited at Cu concentrations above 150 mg·kg^−1^ in soils. Gharbi et al. [4] found that lettuce (*Lactuca sativa*) and spinach (*Spinacia oleracea*) appeared chlorotic when it was exposed to concentrations of Cu ranging from 250 to 1000 mg·kg^−1^ in sandy loam soils. Zhou et al. [5] reported that the presence of external soil Cu (100 mg·kg^−1^) obviously reduced the biomass of 17 bok choy (*Brassica chinensis* L.) cultivars more or less in the range of 25–62.7%. However, Guo et al. [6] indicated that Cu toxicity varied with the soil properties.

Previous studies [3,7] have used total concentration as a regulatory criterion to assess the toxicity of heavy metals in soils, but ignored the mobile and available distinction among different heavy metal forms. There is evidence that soil properties are important factors affecting the chemical forms of heavy metals in soil [8]. For different forms of heavy metals in soils, the water-soluble plus exchangeable fractions are considered to be readily available, whereas the specific adsorbed fraction, which can be extracted by powerful chelating reagents, is potentially available and fractions bound to carbonates, iron (aluminum)-manganese (Fe(Al)-Mn) oxides, organic matter, and minerals are generally not easily taken up by plants [9,10]. Therefore, models incorporating soil properties and heavy metal availability are more reasonable for predicting phytotoxicity. Previous studies indicated that soil pH, organic carbon content (OC), cation exchange capacity (CEC), and soil texture were important factors for establishing phytotoxicity prediction models [11,12,13].

In soil investigations, the most frequently used method to evaluate availability is ethylenediaminetetraacetic acid (EDTA) extraction [14]. According to Sun et al. [15], the formation constant (log K) for 1:1 Cu-EDTA complexes at an ionic strength of 0.01 was 19.7, which was more than those for Ca-EDTA (11.6), Mn-EDTA (14.8), and Al-EDTA (16.5), indicating relatively greater stability of Cu-EDTA in soil. The EDTA-extraction method not only considers the more reactive fractions (water-soluble, exchangeable, and specific adsorbed heavy metals) in heavy metal toxicity prediction, but also accounts for natural background concentrations in environmental risk assessment. Struijs et al. [16] pointed out that environmental risk limits for heavy metals should take into account natural background concentrations and be based on available fractions rather than total concentrations, and they developed the added risk approach to address these issues. In the Netherlands, the maximum permissible concentration, which is used for generic hazard assessment, was recommended as the sum of the available background concentration and the maximum permissible addition [17,18]. The EDTA-extractable concentration, which was applied to indicate the sum of available background and added metals concentrations, solved the question of how to define the available fraction of background concentration.

In the present study, three sequential EDTA-extractions were used to assess the extractability of Cu in order to avoid the effects of total concentration and one-step extraction. Based on EDTA-extractable concentration, the Cu phytotoxicity thresholds for tomato and bok choy biomass were calculated in 17 Chinese soils. Moreover, incorporating soil properties, the EDTA-extractable Cu dose–toxicity relationships for tomato and bok choy biomass were established.

## 2. Materials and Methods 

### 2.1. Soil Samples

Soil samples were collected from 17 sites, which were representative of the major soil types and the distributions of soil pH and organic matter content of Chinese agricultural soils based on the second soil survey of 1982–1994. The detailed soil properties and treatments have been previously described by Li et al. [19]. The ranges of soil properties were as follows (Table 1): pH 4.93–9.80, electrical conductivity 5.7–888 μS∙cm^−1^, CEC 6.36–33.59 cmol^+^∙kg^−1^, OC 0.60–4.28%, CaCO_3_ 0.5–8.92%, oxalate extractable Mn (OX-Mn) 33–451 mg∙kg^−1^, citrate dithionate extractable Mn (CD-Mn) 48–574 mg∙kg^−1^, and citrate dithionate extractable iron (CD-Fe) 3729–83,920 mg∙kg^−1^.

Air-dried soils (0–20 cm) were sieved to <2 mm and spiked with Cu (as CuCl_2_ in deionized water) at eight rates including control treatment: 12.5, 25, 50, 100, 200, 400, and 800 mg∙kg^−1^ for soils with pH < 5; 25, 50, 100, 200, 400, 800, and 1600 mg∙kg^−1^ for soils with pH 5–7; and 37.5, 75, 150, 300, 600, 1200, and 2400 mg∙kg^−1^ for soils with pH > 7. Soil samples were then divided into two batches: one for unleached and one for leached treatments. The unleached soils were incubated for 2 d at 100% water-holding capacity and then air-dried at 25 °C. Subsamples were leached with artificial rainwater to overcome potential salinity effects and to simulate natural precipitation [20]. The artificial rainwater consisted of 5 × 10^−4^ mol∙L^−1^ CaCl_2_, 5 × 10^−4^ mol∙L^−1^ Ca(NO_3_)_2_, 5 × 10^−4^ mol∙L^−1^ MgCl_2_, 10^−4^ mol∙L^−1^ Na_2_SO_4_, and 10^−4^ mol∙L^−1^ KCl (pH 5.9). Similar to the unleached soil samples, the leached soil samples were air-dried at 2 5 °C, sieved to <2 mm using a plastic mesh, and stored for <2 months before the plant assay was conducted.

### 2.2. EDTA-Extractability of Cu in 17 Chinese Soils

The EDTA-extractability of Cu in 17 Chinese soils was determined using Na_2_-EDTA. Briefly, soil samples (5 ± 0.01 g, air-dried, <2 mm) were shaken with 25 mL of 0.05 mol∙L^−1^ Na_2_-EDTA in 50 mL polypropylene centrifuge tubes for 2 h at room temperature (20 °C) in a reciprocating shaker. The soil suspensions were then centrifuged at 2000 g for 20 min, after which the supernatant was decanted and filtered through 0.45-μm filter paper. A fresh batch of 25 mL of Na_2_-EDTA was then added to the residue to continue the extraction. The extraction steps were repeated three times. The filtrate was measured using atomic absorption spectroscopy (ZEEnit 700, Analytik Jena AG, Germany) to determine the EDTA-extractable Cu concentration in soils.

### 2.3. Phytotoxicity Based on EDTA-Extractable Cu in 17 Chinese Soils

Tomato (*Lycopersicon esculentum* ‘Meifen No. 1’), and bok choy (*Brassica rapa* var. *chinensis* ‘Susheng 28’) were used to test the phytotoxicity of Cu in soils by biomass bioassays, according to the International Organization for Standardization [25]. Tomato and bok choy biomass were determined by cultivation in a growth chamber with 25 ± 3 °C day and 20 ± 3 °C night temperatures. The soil moisture content was maintained in the range of 75–90% of field capacity using deionized water throughout the growth period. Tomato and bok choy shoots were cut just above the soil surface, washed with deionized water, then dried at 65 °C for 48 h and dry biomass was weighed.

### 2.4. Phytotoxicity Model Incorporating Soil Properties and EDTA-Extractable Cu Concentration

The phytotoxicity thresholds were calculated as the effective concentrations of EDTA-extractable Cu concentrations inhibiting 10% and 50% of plant growth (EC10-EDTA and EC50-EDTA, respectively). The EC10-EDTA and EC50-EDTA values, along with their 95% confidence intervals, were derived from the fitted dose–response model [26] in Microsoft Excel (Microsoft, Redmond, WA, USA):

(1) $$Y=\frac{Y_{0}}{1+e^{b\left(X-a\right)}}$$

where *Y* is relative plant growth (%), *X* is the log_10_ of the effective concentrations of EDTA-extractable Cu (mg∙kg^−1^), *Y*_0_ is the undisturbed plant growth level, *a* is the log_10_ of EC50-EDTA value (mg∙kg^−1^), and *b* is a curve-fitting parameter indicating the rate of increase of inhibition with increasing concentrations around the EC50-EDTA concentration. Based on the dose–response model [26], the ratio of EC50-EDTA to EC10-EDTA could be closely related to parameter *b*:

(2) $$\frac{\mathrm{EC}50-\mathrm{EDTA}}{\mathrm{EC}10-\mathrm{EDTA}}=10^{\left(-\frac{2.2}{b}\right)}$$

Parameter *b*, which is essential to the shape of the dose–response curve, is closely related to species sensitivity and Cu availability in soils. Previous studies [19,27] showed that the logEC50 of Cu in soils could be predicted by models incorporating soil pH, logOC, and logCEC. Therefore, parameters *b* and *a* were defined as follows:

(3) b=f(pH,CEC,Clay,OXMn,…)

(4) $$a=K_{1}+K_{2}\times pH+K_{3}\times logOC+K_{4}\times logCEC$$

where *K*_1_*–K*_4_ are fitting coefficients of the linear regression relationship between *a* or *b* and soil properties. Based on Equations (3) and (4), Equation (1) can be expressed:

(5) $$Y=\frac{Y_{0}}{1+e^{f(pH,CEC,Clay,OX-Mn,\ldots )\times \left(X-K_{1}+K_{2}\times pH+K_{3}\times logOC+K_{4}\times logCEC\right)}}$$

in which parameters *Y*_0_ and *K*_1_–*K*_4_ can be estimated by regression analysis, conducted using the Solver Function of Microsoft Excel 2010. The parameters in the model were optimized by minimizing the sum of the square of the residual variation of the data points from the model. Relationships were deemed significant at *p* ≤ 0.05.

## 3. Results and Discussion

### 3.1. Phytotoxicity Thresholds Based on EDTA-Extractable Cu for Tomato and Bok Choy

**Figure 1:**
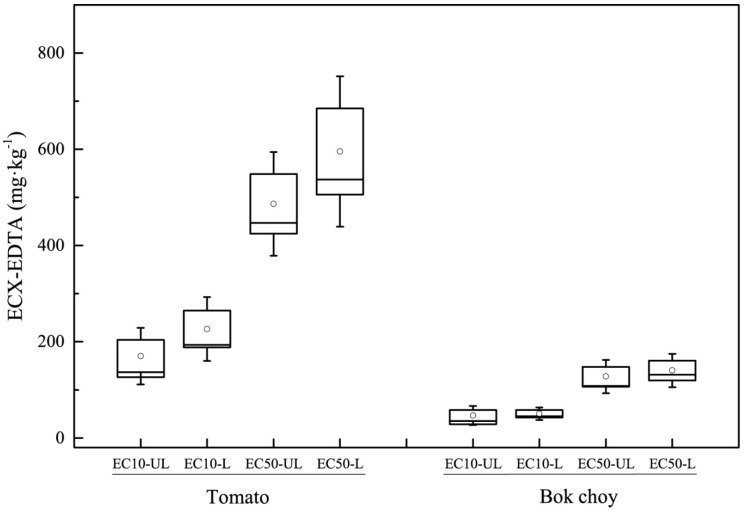
Ranges and variation of Cu phytotoxicity thresholds for tomato and bok choy growth based on EDTA-extractable concentrations in 17 Chinese soils. L = leached soils, UL = unleached soils; ECX-EDTA = effective concentration of EDTA extractable Cu that decreases plant growth by a user-defined percentage; EC10-EDTA = EDTA extractable Cu concentration in soils causing a 10% inhibition in plant growth; EC50-EDTA = EDTA extractable Cu concentration in soils causing a 50% inhibition in plant growth; The bars (Ι) = 10–90% confidence interval; the half box length = standard error; the line in the box = median; ○ = average.

The ranges and variations of Cu phytotoxicity thresholds based on the EDTA-extraction (EC10-EDTA and EC50-EDTA) for tomato and bok choy biomass are shown in Figure 1. Tomato biomass showed no significant difference for EC10-EDTA and EC50-EDTA between unleached and leached soils, with EC10-EDTA values ranging within 32.4–608.8 mg∙kg^−1^ and those of EC50-EDTA 81.7–1398.6 mg∙kg^−1^. The leaching factors, which were defined as the ratio of leached phytotoxicity threshold (EC10-EDTA or EC50-EDTA) to the corresponding unleached phytotoxicity threshold, were 1.51 ± 0.20 and 1.28 ± 0.10, respectively, similar to those reported by Li et al. [27] of 1.54 ± 0.62 and 1.37 ± 0.22, respectively, based on total Cu concentration. The results showed that there was the same influence of leaching on Cu phytotoxicity for tomato based on either available or total concentration.

For bok choy, the EC10-EDTA and EC50-EDTA values ranged within 4.5–148.5 and 33.1–290.3 mg∙kg^−1^, respectively, in the 17 Chinese soils without leaching. In leached soils, excluding Hunan soil, for which an appropriate dose–response model could not be fitted, the EC10-EDTA and EC50-EDTA values ranged within 11.1–120.7 and 22.1–328.6 mg∙kg^−1^ for bok choy, respectively. In both unleached and leached soils, the EC10-EDTA and EC50-EDTA for bok choy were significantly higher than those for tomato biomass, indicating that bok choy was more sensitive to Cu phytotoxicity than tomato. According to Wang et al. [28], bok choy biomass is more sensitive to Cu phytotoxicity than tomato, which fell within the lower position of the species sensitivity distribution. It is likely that Cu accumulation in tissues causes inhibition of growth and reduction in the production of biomass as a general response of metal with a large number of physiological and biochemical processes [29]. As previous studies [30,31] have reported, toxic metals are easily accumulated in leafy vegetables as compared to grain or fruit crops, and most of these plants, especially *Brassicaceae*, grow slowly and have very low biomass productions [29].

### 3.2. Relationship Between Cu Phytotoxicity Thresholds Based on EDTA-Extractable Concentration and Soil Properties

The significant simple and multiple linear regression models to predict Cu phytotoxicity thresholds based on EDTA-extractable concentration (EC10-EDTA or EC50-EDTA) in relation to soil properties are presented in Table 2. For tomato, logarithms of CD-Mn and CD-Fe were found to be the best single factors in predicting EC10-EDTA and EC50-EDTA in unleached soils. According to Li et al. [27], based on total Cu concentration, there were poor single regression relationships between logEC50 and soil properties for unleached soil. Incorporating soil CEC into the regression model for unleached soils increased the *R*^2^ from 0.510 to 0.677 for EC10-EDTA, but for EC50-EDTA including other soil properties did not improve the regression model. Furthermore, when CD-Mn, CEC, and CaCO_3_ were incorporated into the regression model, multiple linear regressions were further improved, with *R*^2^ = 0.852 for EC10-EDTA, indicating that soil properties could explain 85.2% of the variance of log EC10-EDTA. The Fe(Mn) oxides in soils decreased the EDTA-extractability of Cu in soils [32] so that the phytotoxicity thresholds based on EDTA-extractable Cu decreased. Therefore, significant negative relationships between EDTA extractable Cu phytotoxicity thresholds and CD-Mn or CD-Fe were shown in the phytotoxicity predictive models. According to an analysis of soil solution by Li et al. [33], leaching removed water-soluble Cu and increased soil solution pH by up to 0.75 units because soils had a net negative charge. Therefore, both the form of Cu present in soil and soil properties were influenced by leaching treatment. After leaching treatment, the OC and pH became dominant factors in predicting EC10-EDTA and EC50-EDTA—with OC, as the most important single factor, explaining 51.5% and 60.1% of the variance for tomato, respectively.

A comparison of Cu phytotoxicity thresholds showed that bok choy was more sensitive to Cu than tomato. For bok choy, soil OC was the most important factor, consistent with previous results [27], and predicted Cu phytotoxicity thresholds in both unleached and leached soils, explaining 61.7–79.8% and 58.5–75.7% of the variance of EC10-EDTA and EC50-EDTA, respectively. Incorporating CaCO_3_ into regression models increased the *R*^2^ from 0.757 to 0.864 and from 0.585 to 0.771 for EC50-EDTA in unleached and leached soils, respectively. Because of the weaker effect of leaching on Cu phytotoxicity for bok choy [27], the factors affecting Cu phytotoxicity thresholds of bok choy were the same with and without leaching treatment.

### 3.3. Phytotoxicity Predictive Model of EDTA-Extractable Cu for Tomato and Bok Choy

The phytotoxicity thresholds (EC10-EDTA or EC50-EDTA) indicating the degree of Cu phytotoxicity were calculated by dose–response relationships between relative biomass of bok choy or tomato and EDTA-extractable Cu concentrations in soils. According to the description of dose–response curves by Haanstra et al. [26] (Equation (1)), *b* is a slope parameter indicating the inhibition rate with increasing concentrations around the EC50. Additionally, the environmental conditions in the soil not only influenced *b* but also *a*. Therefore, a simple linear correlation analysis was used to determine any significant relationship between soil properties and *b* or *a* in dose–response models. Based on minimizing the sum of the square of the residual variation of the data points from the models, the *b* and *a* incorporating soil properties are shown in Table 3.

**Figure 2:**
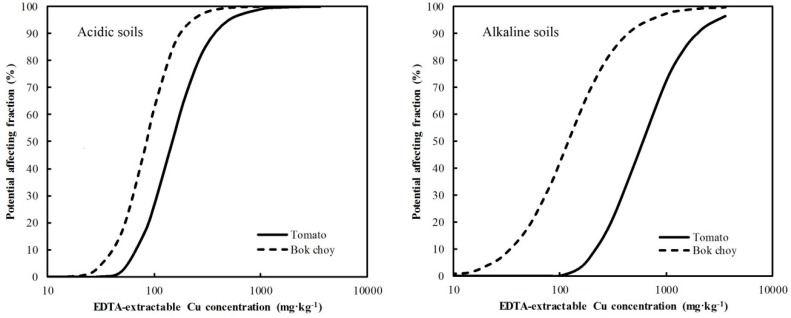
The EDTA-extractable Cu dose–toxicity curves for tomato and bok choy in acidic (pH 5) and alkaline (pH 8) soils (OC was set at 1%, CEC at 20 cmol^+^∙kg^−1^, and OX-Mn at 200 mg∙kg^−1^).

The results of a simple linear correlation analysis showed that the most important factors affecting *a* were soil pH, log OC, and log CEC for tomato and bok choy biomass in soils. However, for different phytotoxicity endpoints, the important factors affecting *b* differed. Specifically, soil pH and OX-Mn were the most important factors for tomato biomass, but soil pH and clay content were most important for bok choy biomass. The absolute values of *b* in the dose–response relationship are independent of the physiological structure of plants, but dependent on the modes of action on the biological target [34]. Moreover, Wang et al. [28] found that soil properties significantly affected *b* values, but *b* values among plant species in the same soil did not significantly differ. Therefore, because of the different action modes between plants and Cu in soil, the dose–response curve shapes differed. Both for tomato and bok choy, the soil pH was one important factor affecting *b*; based on Equation (2), the ratio of EC50-EDTA to EC10-EDTA depended on *b*, so the relationship between EC50-EDTA and EC10-EDTA was related to soil pH. This result was similar to the reported quantitative relationship of different ecotoxicity thresholds for Cu [28], (EC10 = 0.422 EC50–24.059 pH + 150.454, *R*^2^ = 0.73, *p* < 0.05)—when soil pH increased, the ratio of EC10 to EC50 increased and *b* increased. As soil pH increased, the curve became more gradual; therefore, the effect of increasing Cu dose on the toxicity response of plants was less in alkaline than acidic soils [35]. When incorporating the factors affecting *a* and *b*, the soil properties and EDTA-extractable Cu dose explained >90% of the variation of the toxicity response. The relationships between potential affecting fraction of Cu and EDTA-extractable Cu concentration based on the new dose–response curve (Table 3) are shown in Figure 2 for OC at 1%, CEC at 20 cmol^+^∙kg^−1^, and OX-Mn at 200 mg∙kg^−1^, indicating that at the increased EDTA-extractable Cu concentration, there was a higher potential effect on bok choy than tomato—thus bok choy was clearly more sensitive than tomato. The difference in sensitivity between bok choy and tomato was greater in alkaline than acidic soil. When the EDTA-extractable Cu concentration was >100 mg∙kg^−1^, the same EDTA-extractable Cu concentration had less potential phytotoxic effect in alkaline soils for both bok choy and tomato. In other words, a limited EDTA-extractable Cu concentration leading to the same potential phytotoxicity effect was higher in alkaline than acidic soils. The limited value based on EDTA-extractable Cu concentration for a specific plant species could be calculated by the equation $X=\frac{\ln\left(Y_{0}-Y\right)-\ln Y}{b}+a$, which incorporated soil properties (Table 3). Therefore, the phytotoxicity endpoint and soil properties were critical factors in developing a reasonable remediation strategy for contaminated soils.

**Figure 3:**
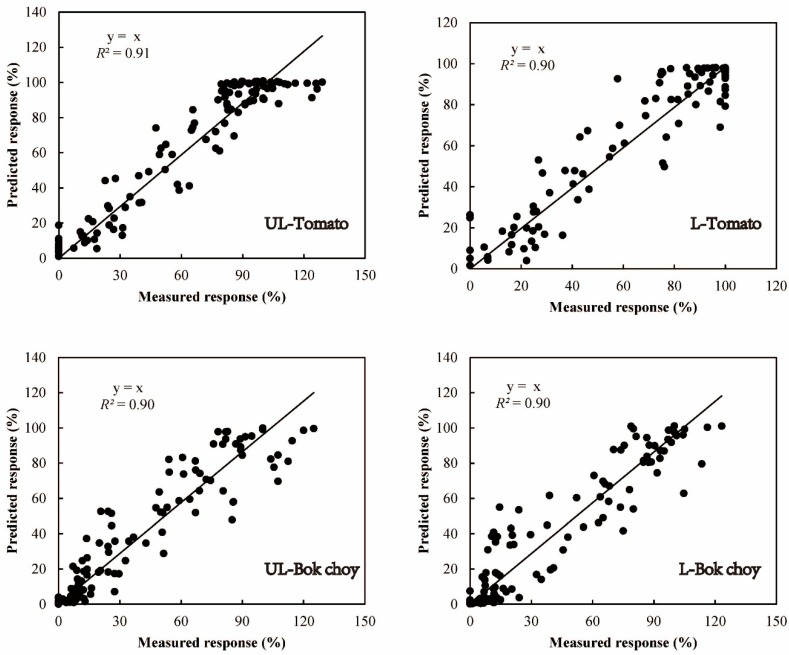
Measured versus predicted toxicities for tomato and bok choy biomass based on EDTA-extractable dose–toxicity relationships (see Table 3). UL and L represent unleached and leached treatments, respectively.

The model prediction effect for tomato biomass (Figure 3) showed that the predicted response reached 100% as the EDTA-extractable Cu concentration increased; however, compared with tomato control biomass, the measured response reached 129% when EDTA-extractable Cu concentration was 23.65 mg∙kg^−1^ because of the hormesis of tomato [27,36]. Additionally, an evaluation of the tomato biomass revealed that the EDTA-extractable Cu dose–toxicity relationship was predicted better in unleached than leached soils. Because of the weaker effect of leaching on Cu phytotoxicity for bok choy biomass, the predicted effectiveness did not differ between leached and unleached treatments.

## 4. Conclusions

In the present study, phytotoxicity thresholds of Cu based on the concentrations of sequential EDTA-extractable Cu across a wide range of Chinese soils varied 18.8-fold for tomato and 32.9-fold for bok choy. Incorporating soil properties, the prediction model of logEC10-EDTA and logEC50-EDTA showed that CD-Fe was the most dominant factor (*R*^2^ = 0.684), followed by CD-Mn (*R*^2^ = 0.510), for tomato, and OC was the most dominant factor (*R*^2^ = 0.798) for bok choy. Furthermore, predictive models of dose–toxicity relationships for a wide range of soils based on the EDTA-extractable Cu were established in order to predict the phytotoxicity response of tomato and bok choy in Chinese soils (*R*^2^ ≥ 0.90). Based on minimizing the sum of the square of the residual variation of the data points from the models, parameters *a* and *b* were found to be functions of soil properties—this showed that soil pH, logOC, and logCEC were the most important factors to *a* for tomato and bok choy biomass in soils. Because of the different action modes between plant species and Cu in soil, the factors affecting *b* differed between tomato and bok choy. When incorporating factors that affected *a* and *b*, the soil properties and EDTA-extractable Cu dose explained >90% of the variation of the toxicity responses.

## Figures and Tables

**Table 1:** Main soil properties of 17 Chinese sites used in the study.

Site	pH ^1^ (1:5)	EC (μS∙cm^−1^)	CEC ^2^ (cmol^+^∙kg^−1^)	OC ^3^ (%)	CaCO_3_ (%)	OX ^4^-Mn (mg∙kg^−1^)	CD ^5^-Mn (mg∙kg^−1^)	CD ^5^-Fe (mg∙kg^−1^)
Haikou	4.93	110.8	8.75	1.5	<0.5	200	574	83,920
Qiyang	5.31	74.1	7.47	0.9	<0.5	294	422	26,154
Hailun	6.56	153	33.6	3	<0.5	451	396	6559
Jiaxing	6.7	158.8	19.3	1.4	<0.5	261	297	10,824
Hangzhou	6.8	203.3	12.83	2.5	<0.5	135	153	8413
Chongqing	7.12	71	22.3	1	<0.5	283	315	7727
Guangzhou	7.27	136.7	8.3	1.5	0.15	33	48	11,411
Lingshan	7.48	92.5	22.6	4.3	4.27	267	276	6950
Hulunber	7.66	888	22.7	2.7	0.27	307	322	5259
Gongzhuling	7.82	146.9	28.7	2.2	0.27	387	366	6932
Shijiazhuang	8.19	302	11.7	1	3.84	222	261	7544
Urumchi	8.72	226.5	10.3	0.9	5.08	251	305	4795
Yangling	8.83	83.2	8.46	0.6	8.92	288	350	7193
Langfang	8.84	5.7	6.36	0.6	2.42	74	112	3729
Zhangye	8.86	108.7	8.08	1	7.75	121	166	4289
Zhengzhou	8.86	151.8	8.5	1.6	0.15	233	331	8356
Dezhou	8.9	111.8	8.33	0.7	6.17	145	219	4965

^1^ Measured in deionized water (soil: solution ratio 1:5). ^2^ Cation exchange capacity, determined using ammonium chloride method. ^3^ Organic carbon content, determined as the difference between total carbon [21] and inorganic carbon content [22]. ^4^ Oxalate extractable metal [23]. ^5^ Citrate dithionate extractable metal [24].

**Table 2:** Simple and multiple linear regressions between phytotoxicity thresholds based on EDTA-extractable Cu and selected soil properties for tomato and bok choy biomass.

Plant	Treatment	Regression Equation	*R* ^2^	*p*		
Tomato	Unleached	log EC10-EDTA = 3.588 − 0.607 log CD-Mn	0.51	0.036		
	(*n* = 17)	log EC10-EDTA = 3.377 − 0.803 log CD-Mn + 0.617 log CEC	0.677	0.006	0.04	
		log EC10-EDTA = 3.305 − 0.990 log CD-Mn + 0.971 log CEC + 0.057 CaCO_3_	0.852	<0.001	0.001	0.003
		log EC50-EDTA = 5.104 − 0.636 log CD-Fe	0.684	0.002		
	Leached	log EC10-EDTA = 2.139 + 0.725 log OC	0.515	0.034		
	(*n* = 17)	log EC10-EDTA = 0.870 + 1.015 log OC + 0.162 pH	0.737	0.002	0.011	
		log EC50-EDTA = 4.931 − 0.577 log CD-Fe	0.523	0.031		
		log EC50-EDTA = 2.552 + 0.844 log OC	0.601	0.011		
		log EC50-EDTA = 1.124 + 1.169 log OC + 0.183 pH	0.845	<0.001	0.002	
Bok choy	Unleached	log EC10-EDTA = 1.286 + 1.377 log OC	0.798	<0.001		
	(*n* = 17)	log EC50-EDTA = 1.910 + 0.838 log OC	0.757	<0.001		
		log EC50-EDTA = 1.775 + 1.092 log OC + 0.043 CaCO_3_	0.864	<0.001	0.008	
	Leached	log EC10-EDTA = 1.526 + 0.690 log OC	0.617	0.011		
	(*n* = 17)	log EC50-EDTA = 1.981 + 0.651 log OC	0.585	0.017		
		log EC50-EDTA = 1.808 + 0.960 log OC + 0.054 CaCO_3_	0.771	0.001	0.014	

EC10-EDTA and EC50-EDTA, EDTA-extractable Cu concentration causing 10% and 50% inhibition of tomato or bok choy biomass, respectively; CD-Mn, citrate dithionate extractable Mn concentration; CEC, effective cation exchangeable capacity; CD-Fe, citrate dithionate extractable Fe concentration; OC, organic carbon content; *p*, significance level of the factors included in the regression equations.

**Table 3:** The parameters in the EDTA-extractable Cu dose–toxicity relationships incorporating soil properties for tomato and bok choy biomass in unleached and leached soils based on Equation (1): $Y=\frac{Y_{0}}{1+e^{b\left(X-a\right)}}$ or $X=\frac{\ln\left(Y_{0}-Y\right)-\ln Y}{b}+a$.

Phytotoxicity Endpoints	Treatment	*Y*_0_	*a*	*b*	*R* ^2^	*p*
Tomato	Unleached	100.82	*a* = 0.63 + 0.21 pH + 0.54 logOC + 0.28 logCEC	*b* = 0.63 pH + 0.002 OX-Mn − 9.59	0.92	<0.01
	Leached	106.30	*a* = 0.72 + 0.20 pH + 0.71 logOC + 0.33 logCEC	*b* = 0.43 pH + 0.007 OX-Mn − 6.21	0.91	<0.01
Bok choy	Unleached	99.93	*a* = 1.43 + 0.07 pH + 0.99 logOC − 0.02 logCEC	*b* = 1.13 pH − 12.61	0.91	<0.01
	Leached	101.09	*a* = 1.61 + 0.05 pH + 0.77 logOC + 0.05 logCEC	*b* = 0.98 pH − 11.75	0.90	<0.01

*Y*, relative plant growth (%) indicating the potential affecting fraction of Cu on tomato and bok choy growth; *X*, log10 of the effective EDTA-extractable Cu concentration; *Y*_0_, curve fitting parameter indicating undisturbed plant growth level; *a*, curve-fitting parameter indicating log10 of EC50-EDTA value; *b*, curve-fitting parameter indicating the rate of increase of inhibition with increasing EDTA-extractable Cu concentrations.
